# h-Profile plots for the discovery and exploration of patterns in gene expression data with an application to time course data

**DOI:** 10.1186/1471-2105-8-486

**Published:** 2007-12-20

**Authors:** Yvonne E Pittelkow, Susan R Wilson

**Affiliations:** 1 Mathematical Sciences Institute, Australian National University, Building 27, Canberra, ACT 0200, Australia

## Abstract

**Background:**

An ever increasing number of techniques are being used to find genes with similar profiles from microarray studies. Visualization of gene expression profiles can aid this process, potentially contributing to the identification of co-regulated genes and gene function as well as network development.

**Results:**

We introduce the h-Profile plot to display gene expression profiles. Thumbnail versions of plots of gene expression profiles are plotted at coordinates such that profiles of similar shape are located in the same sector, with decreasing variance towards the origin. Negatively correlated profiles can easily be identified. A new method for selecting genes with fixed periodicity, but different phase and amplitude is described and used to demonstrate the use of the plots on cell cycle data.

**Conclusion:**

Visualization tools for gene expression data are important and h-profile plots provide a timely contribution to the field. They allow the simultaneous visualization of many gene expression profiles and can be used for the identification of genes with similar or reversed profiles, the foundation step in many analyses.

## Background

In gene expression (or transcript profiling) experiments, gene expression is often compared under different experimental perturbations, such as between different genotypes, cell lines, diseased and healthy subjects or under different treatment conditions. Another, complementary approach, which is useful for understanding the dynamics of altered gene expression, is via time course data, where gene expression is measured at different time intervals, usually under the same experimental conditions.

Genes with similar expression profiles, i.e. co-expressed genes are potentially co-regulated and finding these genes is often a first step in inferring gene function and in developing gene networks.

An ever increasing number of techniques are being used to find genes with similar profiles from microarray studies. Many of these methods are adapted from statistics and/or machine learning. Visualizing the gene expression would aid this process but is a challenge because of the number of genes involved and the complexity of organizing the patterns.

In this paper we describe methods which can be used for visualizing gene expression profiles. The term, gene expression profile, is used differently by different authors but essentially it refers to gene expression values (or their transformations) on all arrays for a given gene, or a summary of the gene's expression values, such as the mean, in different groups or classes of arrays. Line plots of the gene expression measurements, or summaries of gene expression in subsets of the arrays, are arguably the most common plot used for visualization gene expression data. However any plots, icons, or glyphs such as Chernoff faces, castles, trees, survival curves which capture some aspect of a gene's expression profile can be used. See for example Chambers *et al. *[1] and references therein for further ideas. We use the term 'profile plot' to describe such plots.

The approach taken in this paper is straightforward. 'Thumbnail' versions of the plot for the genes selected to be displayed are plotted at the coordinates of the h-plot [2,3]. The word thumbnail is used in the sense of a reduced image of a graphic, utilized in order to view multiple images on a visual device simultaneously. They make it easier to visually scan and recognize broad groupings of profiles and unexpected profiles. Details are of course sacrificed because of the small size but often it is vital to obtain a broad view of the profiles, followed by more detailed plots.

There are a number of decisions which the analyst needs to make. They include the choice of plot to describe the gene profile, which genes are to be displayed, and the placement of the thumbnails in the display. Preprocessing of the gene expression measurements, such as scaling, normalization and transformation can have a profound effect on the visual effect of the profile and these decisions also need to be considered carefully.

In this paper we focus on the time series graph where lines join the gene expression measurements (transformed or otherwise) in order of time. Line graphs, where lines connect mean gene expression in different classes or time intervals are also useful.

Other plots to use as thumbnails for time series data, include the autocorrelation function (acf), periodogram, spectral plots and fitted values from a statistical model. The acf plot, periodogram and spectral plots exhibit different characteristics for data which follows a pure random process to data where there is a periodic signature. Wichert *et al. *[4] proposed a version of the periodogram as a graphical exploratory device for microarray data.

There have been a number of interesting approaches to the identification of periodically expressed genes, but for reasons of space we cannot describe them all here. An early application was described in Spellman *et al. *[5]. They combined Fourier analysis with SVD. A recent example is given by Luan and Li [6] who use linear combinations of B-splines basis to identify genes which follow the same periodic expression pattern, subject to horizontal shift and/or different amplitudes to a set of guide genes. A number of authors have used regression models. For example Liu *et al. *[7] used quadratic regression and Venezia *et al. *[8] used orthogonal polynomial regression models and Liu *et al. *[9] use a circular regression. Some selection methods have been based on the periodogram. For example Wichert *et al *[4] use an exact test applied to periodograms, Earl *et al. *[10] used the Lomb-Scargle periodogram.

In 2005 de Lichtenberg *et al. *[11] compared a number of different methods used to identify cell cycle-regulated genes. They show that some new and more sophisticated methods for selecting cell cycle-regulated genes performed worse than the analysis published with the original data sets. Also they showed that the genes selected visually by Cho *et al. *on two of their benchmark data sets 'was remarkable'. They conclude that methods need to accommodate both the periodicity and the magnitude of regulation to be biologically relevant.

In this paper we introduce a new selection method which provides estimates of amplitude and phase and show that it performs well. We demonstrate the use of the h-profile plot on genes selected by our method and compare the selections with genes previously identified by Cho *et al. *[12] and de Lichtenberg *et al. *[11].

## Results

### Coordinates

Let **Z **be the gene centered matrix, with *N*_*g *_genes in the columns and *N*_*c *_microarrays in the rows. Further, let the SVD of **Z**, be

**Z **= **U Λ V**^*T*^,

where **U**, size *N*_*c *_by *N*_*c*_, and **V**^*T*^, size *N*_*g *_by *N*_*g*_, are orthogonal matrices such that **U**^*T*^**U **= **I **and **V**^*T *^**V **= **I**. The notation **I **is used to denote a conformable identity matrix.

Then the coordinates of the h-profile plot in k dimensions, are defined as

$${\hat{G}}_{k}^{T}=\frac{1}{\sqrt{N_{c}-1}}\Lambda_{k}V_{k}^{T},$$

where **U**_*k *_and **V**_*k *_are matrices comprising the first *k *columns of **U **and **V **respectively and **Λ**_*k *_is a sub matrix of **Λ **formed from the first *k *columns and rows of **Λ**. For two dimensional representation *k *= 2.

**Figure 1:**
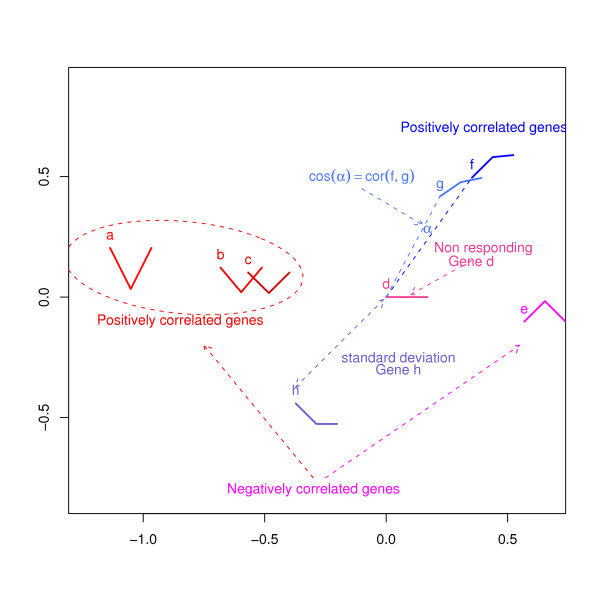
Schema for an h-Profile plot using expression levels or profiles for eight genes, a, ..., h, in three microarrays. The line graphs used for the thumbnail plots have the relative gene expression level on the vertical axis and the horizontal axis has equal intervals, representing the first, second and third microarray respectively.

In the h-profile plots the geometric distance from the origin to the gene points corresponds to an approximation to the standard deviation of individual gene expression. Figure 1 shows a simplified example of an h-profile plot for eight genes from three microarrays. The expression levels or profiles are represented by a thumbnail version of the line graph. For each gene, the horizontal axis of its line graph has equal intervals, representing the first, second and third microarray respectively. Gene a, for example, has equally high expression in the first and third microarray but is relatively down regulated in the second microarray.

In Figure 1 gene a has the highest standard deviation and lies most distant from the origin, (0,0). Gene d which is not changing over the 3 microarrays is placed close to the origin (it has zero standard deviation).

The angular separations between the gene points in the plot are approximations to cosines of correlation between the gene expression profiles. In Figure 1, *α*, the angular separation between genes f and g, is quite small, and therefore the cosine of *α *will be high, indicating, as one might expect from the shape of their profiles, high correlation. Gene h which has a profile of opposite 'shape' to these genes i.e. is highly negatively correlated with these two genes, is placed on the other side of the plot.

Genes a, b and c are also highly positively correlated and lie in the same sector, lying approximately on a line passing through the origin. As can be seen, their profiles are of similar 'shape', but with decreasing 'steepness', i.e. decreasing variance from a to c. Gene e has the reversed profile to gene a and its profile is placed on the opposite side of the plot also lying on the line passing through genes a, b and c and the origin.

In general, genes which lie on a line passing through the origin will be correlated, positive if on the same side and negative if on the other side. The distance between two genes approximates the standard deviation of the difference between the two genes.

It should be noted that when interpreting and comparing h-plots one uses the configuration of the points (or icons) without particular reference to the coordinate axes. The axes are used only for constructing the plots, not for interpreting them as is commonly done in PCA and factor analyses.

Associated with the h-plot is the covariance biplot, where both genes and microarrays are shown in the same plot. The coordinates for the microarrays in the covariance plot are

$$\hat{C}=\sqrt{N_{c}-1}U_{k}.$$

Sometimes *N*_*c *_is used instead of *N*_*c *_- 1 in the equations for the gene and microarray coordinates. The interpretation for the angular distance between the genes in this plot is the same as in the h-plot but additional information is possible using a geometric interpretation of inner products. This biplot is one of the biplots introduced by Gabriel [3] with variants called the covariance biplot [13] and the *GE*-Biplot [14].

It is easy to show that *Z*_*k *_= **C**_*k *_$G_{k}^{T}$, where *Z*_*k *_is a rank *k *approximation to *Z*. Thus in biplots, where the microarrays, *C*_*k*_, and the genes, *G*_*k*_, are plotted on the same graph, the scalar product between the *i*^*th *^row point and *j*^*th *^column point with respect to the origin is approximately equal to the (*i, j*)^*th *^element, *z*_*ij*_, of **Z**, the transformed, mean corrected gene expression matrix used in the biplot.

In the particular biplot used here, called the GE-biplot (Gene Expression Biplot) to differentiate it from the original covariance biplot, the juxtapositions of the gene points to the microarray points provides an approximation of the value of the (transformed) gene expression values on the microarrays. Geometrically, the inner product can be viewed as the product of the signed length of one of the vectors and the length of the projection of the other vector onto it. Thus if a gene point is close to a microarray point then it can be deduced that the gene is relatively up regulated in the microarray represented by the point and if the gene point is on the opposite side of the plot to the microarray point then the gene is relatively down regulated on the microarray. How accurate these deductions are depends on how good the approximation is in the lower ranked space. Two measures of fit, *I*_1 _and *I*_2_, ranging from 0 to 1, are described in the Methods section.

There is a rich interpretation in terms of difference vectors for this biplot which we do not describe here but further information can be found in the methods section and in [3,13] and [14].

With the biplot, using the factorization of the gene centered matrix described here, the distance between two microarray points approximates the Mahalanobis distance standardized in the plane of fit [3] between the microarrays. Mahalanobis distance, also called the standardized or generalized distance, is arguably more appropriate than Euclidean distance for microarray data, as it takes correlation into account.

### Gene Selection for cyclic data

The profiles, and theoretical arguments, suggest that finding genes with a log profile described by

*z *= |*Asin*(2*πBt *+ *L*)|,

is of interest. In this equation *z *is the gene expression (possibly transformed), *A *is the amplitude, *B *corresponds to the period and *L *to the part of the cycle at time zero i.e. related to phase. This model allows the selection of genes with different phases and/or amplitudes.

If *B *= 1 and time *t *is scaled to run from 0 to 1, there are exactly two cycles and the aim is to choose genes whose profiles complete two cycles and have approximately equal amplitude in both cycles. The model

*z *= |*Asin*(2*πt *+ *L*)|,

can be estimated for each gene using non-linear least squares and a scale free estimate of residual error

$$RSS=\sum_{t}{\left((z_{t}-{\hat{z}}_{t}^{*})/\hat{A}\right)}^{2},$$

where ${\hat{z}}_{t}^{*}=|\hat{A}sin(2\pi t+\hat{L})|$ obtained.

To assess fit, using *RSS*, knowledge of the distribution of *RSS *under the null model is required. To obtain this we simulated 1000 data points, y, where *y*_*t *_= |*Asin*(2*πt *+ *L*)| + *ε *and without loss of generality, *A *= 1 and *L *= 0 with *ε *~ *N*(0, $\sigma_{\varepsilon }^{2}$). The average within phase variance across all genes was used as an estimate of *σ*_*ε *_in the simulation.

Different quantiles of the distribution of *RSS *for the simulated data, denoted by $\hat{c}$, can be used to select genes which fit the model. Estimates of *RSS*_*g *_for all genes, g, were calculated and compared to $\hat{c}$. All genes, where $R\hat{S}S_{g}\leq \hat{c}$ were selected as being compatible with the two cycle model. Different values of $\hat{c}$ select genes with more or less compatability with the model.

Estimates of *A *from these fits provide information on the extent of gene expression change (amplitude), and the value of *t *when $\hat{z}$ = *A *is an estimate of the time of maximal expression. The intercept parameter *L *determines the expression at the beginning of the cycle. Displays of this function, with *A *= 1 and *B *= 1 for values of 0 ≤ *L *≤ 3 are shown in additional file 1.

### Mitotic Cell Cycle Data

The time-course data set described in Cho *et al. *[12] and available from the website [15] consisted of 6601 gene expression measurements from 17 Affymetrix pairs of chips. The aim of the study [12] was to characterize mRNA transcript levels during the cell cycle of the budding yeast S. *cerevisiae*. Synchronous yeast cultures were arrested in late G1 and the cell cycle re-initiated with cells collected at 10 minute intervals, covering nearly two full cell cycles. The time course was divided into early G1, late G1, S, G2 and M phases based on the size of the buds, the cellular position of the nucleus, and standardization to known transcripts.

In the initial phases of their analysis, Cho *et al*. selected 1348 out of 6565 genes which had a 2 fold difference more than 40 minutes past the point of release from arrest. They visually inspected the profiles for these genes for evidence of periodicity of expression and chose 416 genes with identifiable periodic changes in normalized (but not, apparently, logged) gene expression.

The normalization method used by Cho *et al*. was not described in their paper. However the distributions of gene expression measurements from each microarray were similar, although positively skewed, as is common with untransformed gene expression values. There were however a large number of negative measurements, especially for samples taken at 90 and 100 minutes. In our analyses the negative values were truncated to .01, the data logged using base 2, and finally standardized so that the microarray means are zero and their variance one. This later transformation effectively 'normalizes' the distributions so that the first two moments agree. Control genes were removed leaving a total of 6565 genes. Although not technically correct we will refer to the transformed gene expression as simply gene expression to avoid cumbersome expressions. But it should be kept in mind that the preprocessing can have a large impact on analyses. The sample at time zero, that is immediately after arrest, was eliminated from the following analyses, leaving two microarrays in each phase.

**Figure 2:**
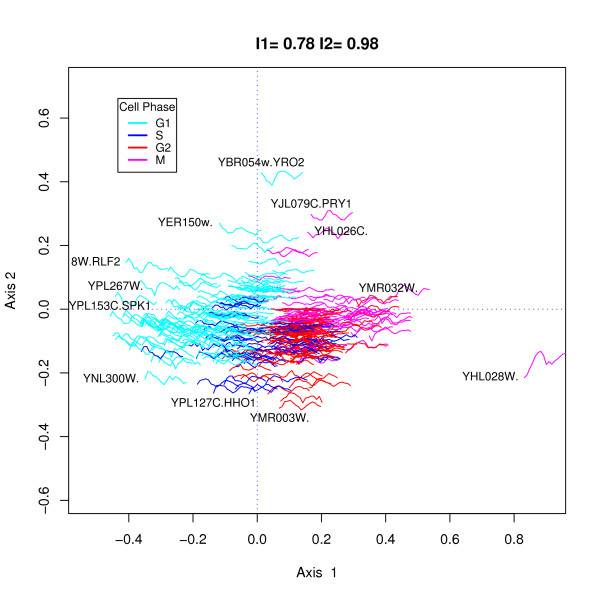
h-Profile plot using 352 genes selected from the Cho *et al *data set using equation 5 with a critical cutoff estimated using the third quartile of the simulated values of *RSS*. The profile plots show a time series plot of the transformed gene  expression values, colored according to the phase in which the maximum gene  expression value occurred. A number of gene profiles on the outer edge of the bulk of the thumbnails are identified, all of which were in de Lichtenberg *et al*'s top 300 list [11] and 9 in the Cho *et al *[12] list. By the geometry of the h-profile plot these genes can be expected to have the highest variance amongst the selected genes and therefore, in the sense of de Lichtenberg *et al*, to be strongly regulated.

Figure 2 shows an h-profile plot of the 352 genes selected using equation 5 with a critical cutoff estimated using the 75^*th *^quantile of the simulated values of *RSS*. Of these selected genes 147 (42%) were also identified by Cho *et al. *[12] (CHO list) and 114 were identified amongst de Lichtenberg *et al*.'s [11] top 300 (L300 list). 92 were identified in both CHO and L300 lists. The thumbnail plot is a line plot connecting the gene expression value at each of the 16 time points and are colored according to the phase at which the maximal gene expression occurred. The fit indices of .78 and .98 are high (maximum value is one) indicating a good but not perfect fit in two dimensions. A number of gene profiles which show greater amplitude are identified for illustrative purposes. All of these genes were in the L300 list and 9 in the CHO list. In practice, h-profile plots are used interactively and genes whose profiles are of interest are identified and investigated further. It is also clear that genes which have their maximal expression in different phases tend to group together although there is some overlap between adjacent phases.

**Figure 3:**
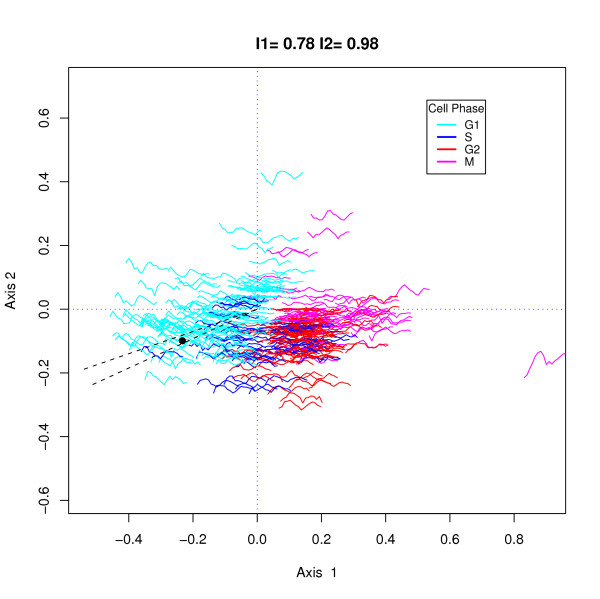
h-profile plot for the 352 genes from the Cho *et al *data selected according to the sin model of equation 5 with the location of gene CLN1, a landmark gene used for late G1 by Cho *et al*. indicated by a black dot. The sector used for selecting genes correlated with CLN1 is delineated by dashed lines. Gene profiles are colored according to the phase in which their maximum expression occurred.

Sometimes, as in Cho *et al. *[12], landmark genes are available which have previously been characterized with respect to a specific cell cycle phase. These genes can be identified, if they are among the selected genes, and their profiles inspected. For demonstration purposes we have chosen to concentrate on two landmark genes identified by Cho *et al*, and illustrate how these plots can be used to identify genes of similar profiles. One could identify them visually using the plots, zooming in if necessary, but, alternatively, since we know that highly correlated genes have small angular distance we can select all genes within a given angle of the landmark gene. Figure 3 shows the same profile plot as in Figure 2 but with the location of the first landmark gene, CLN1, used for late G1 by Cho *et al*., shown by a black dot. The profile is colored cyan which tells us that its maximum expression occurred in one of the samples in the G1 phase. To identify genes with similar shaped profiles, we selected all genes in the sector of the plot between the dashed lines.

**Figure 4:**
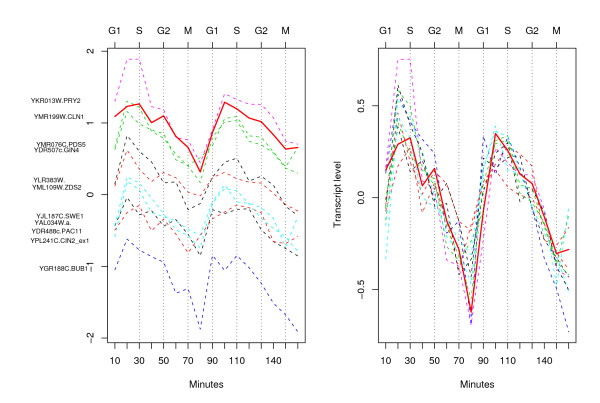
Detailed profiles for the genes which were within the sector shown in the previous h-profile plot. The gene expression values are shown on the vertical axis on the left, and on the right, the mean centered gene expression profiles are shown. The profile of CLN1 is shown as a red solid bold line. All these genes have a correlation of .74 or higher with CLN1. The phase identification on the top axis identifies the first of the two samples which were identified to belong to the phase by Cho *et al *[12].

There were 11 genes located within the sector and their identifications are shown on the left of Figure 4. All these genes had a correlation of .74 or higher with CLN1. The profile of CLN1 is shown as a bold red line. Six of these 11 genes were also in the L300 list and 9 in the CHO list. At this detail one can see that in the first cycle, CLN1 peaked in the S phase (30 minutes) although it peaked in the G1 phase in the second cycle. Most of the profiles peak in G1 and it is clearer in these more detailed plots that they tend to have higher amplitudes in the first cycle than the second. The plot on the right shows the same profiles, with the gene means subtracted, highlighting the consistency in gene expression profiles between these genes.

**Figure 5:**
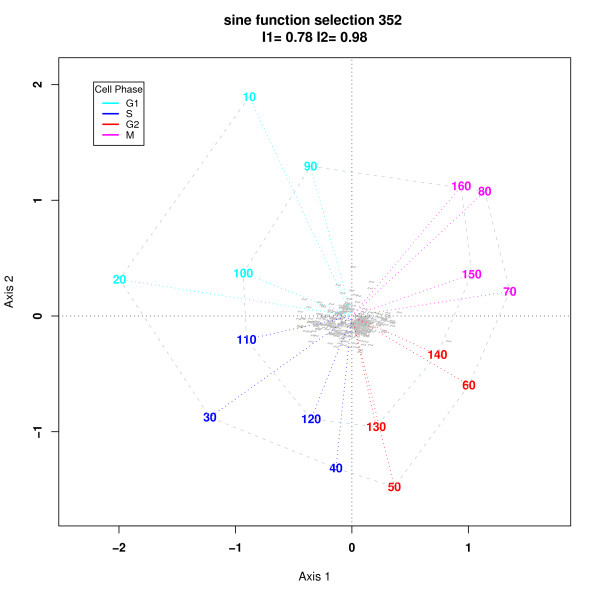
A covariance biplot (GE-Biplot) using the same set of selected genes. The genes are shown as tilde, marking their positions relative to the chip points which are shown as numerals indicating the time in minutes, with colors corresponding to the cell phases as determined by Cho *et al*. The genes in this plot have the same coordinates as in Figure 3. The chips allocated to the differently colored phases appear in the same region and have been joined by a dashed line in time-sequence. The spiral shape of the dashed line confirms that these genes do overall follow a cyclic pattern. *I*_1 _and *I*_2 _shown in the title, are two fit indices with a maximum value of 1, described in methods.

In Figure 5, a covariance biplot (GE-biplot) using the same set of selected genes is shown. The genes are shown as tildes simply marking their positions relative to the chip points which are shown as numerals indicating the time in minutes, with colors corresponding to the cell phases as determined by Cho *et al*. The genes in this covariance biplot have the same coordinates as in h-profile plot of Figures 2 and 3. The chips allocated to the differently colored phases appear in the same region and the strict ordering of the chips in time around the origin indicates strong cyclic behavior in time. The chips have been joined by a dashed line in time-sequence and show a spiral. The distances between the chips for the beginning phases of first and second cycle suggest that the rate of change (log scale) was greater in the early stages of the first cell cycle than in the second. By the end of phase S the genes in the two cycles are beginning to be more similar and by late mitosis (M) the cycles are more or less coinciding in the sense that the gene expression in these genes are the same. These general interpretations are possible because of the geometry of inner products. If the microarray points are close, then in general the gene expression for these selected genes on these chips will also tend to be close.

The difference in gene expression between the two cycles in the early phases has a number of potential causes such as problems with the arrest method, loss of synchronization [16] or temperature induced effects [12]. The profiles shown in Figure 4 also support this interpretation for the selected genes.

We now focus on finding genes with similar profiles to CLB1, the other landmark gene, for the M phase, and used by Cho *et al*. Here, we used the median of the simulated values of fit to define the criteria for selection. 157 genes satisfied this criteria and of these, 79 were also in the CHO list and 57 in the L300 list.

**Figure 6:**
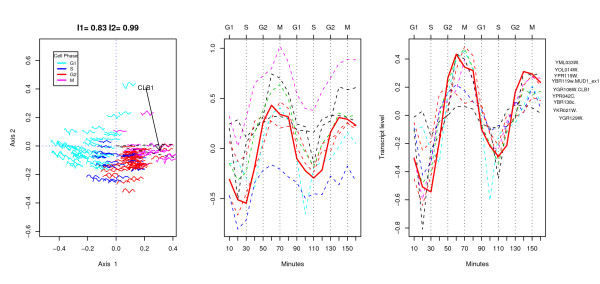
On the left an h-profile plot for 157 genes selected according to the sin model using a more stringent criteria for selection than in the earlier h-profile plots, and with the location of gene CLB1, a landmark gene for the M phase, shown by the arrow. The two right hand plots show the detailed profile plots for the genes located within the sector identified in the h-profile plot on the left. The profiles on the right have been centered so that each gene has zero mean.

Figure 6 shows an h-profile plot on the left for these 157 genes. The fit of the plot (in two dimensions) is very high indicating that for these genes, much of the correlations are captured in the ordination. The location of CLB1 is indicated by an arrow.

The profiles of 9 genes in a sector around CLB1 are shown in the middle and the right of the figure. 6. All of these genes were in the CHO list and 4 in the L300 list. They all peak between 60 and 70 minutes and have a correlation coefficient of .7 or higher with CLB1. Interestingly, CLB1 is a landmark gene for the M phase, but in this data it has peak expression on the arrays taken at 60 minutes and 140 minutes past arrest, which was identified by Cho *et al *to be in the G2 phase. This is not a product of the transformation and scaling of the chips used in this analysis, as this profile is consistent with Figure 4 of Cho *et al. *[12]. In practice one would want to check the profiles of all the landmark genes for phase M and see if this were a consistent pattern for these data. The ability to visualize the profiles allows the researcher to question meaningfully the criteria used for searching and selection.

**Figure 7:**
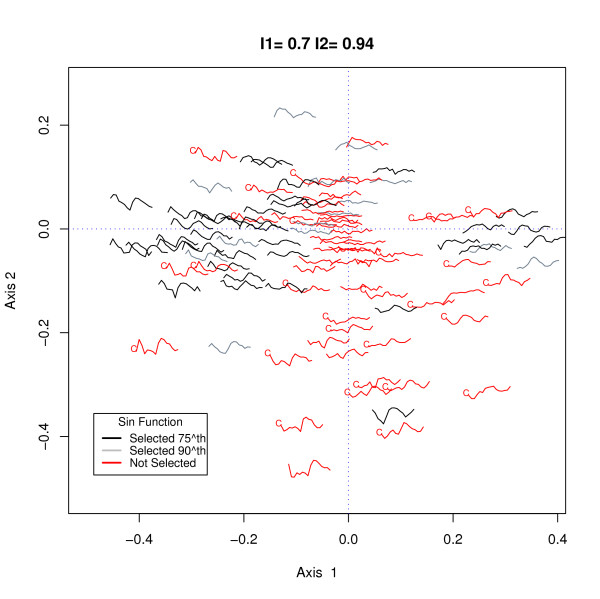
h-profile plot for 104 genes which were in the Benchmark data set, B1, of de Lichtenberg *et al *[11] This list consisted of genes which were previously identified as periodically expressed in small-scale experiments. Profiles which were selected using the 75^*th *^percentile of the simulated data as the critical value are shown in black, and those selected using the 90^*th *^percentile are drawn in grey. Profiles which were not selected but are in the B1 list are shown in red. 28 genes which were identified by Cho *et al *but not by our sin function method are marked by the letter "C".

As a final example we plot the genes which were in the benchmark data set, B1, of de Lichtenberg *et al. *[11]. This list consisted of 113 genes which were previously identified as periodically expressed and used by Spellman *et al. *[5] with some additions by Johansson *et al. *[17]. Our sin selection method found 35.2% of these genes and we use the h-profile plot to understand the reason for the discrepancy. Of the 113 genes we matched 108 and their profiles are shown in an h-profile plot in Figure 7. In this plot profiles which were selected using the 75^*th *^percentile of the simulated data as the critical value are shown in black, and those selected using the 90^*th *^percentile are drawn in grey. Profiles which were not selected but are in the B1 list are shown in red. A reasonably quick appraisal of the red profiles indicates many genes whose profiles do not exhibit a periodic pattern of expression, thus throwing doubt on their status as cell cycle genes. Another possibility is that for some genes which are cell cycle-regulated, smooth periodic functions such as we used, or Fourier based functions as used by others, are not suited. Cho *et al *who did not use a periodic function, relying on visual identification, found 28 additional genes to the ones selected by the use of our sin function. These profiles are marked by the letter "C" in the plot. As expected, many of these additional genes do not appear periodic over the 16 time points. There do not appear to be other common patterns discernible, so we hypothesize that many of these additional genes were selected as a consequence of the fact that Cho *et al *used the profiles only after 40 minutes past arrest to make their selection.

## Discussion

In almost all microarray applications, the analysis starts with performing some transformation on a data matrix, depending on the nature of the data. Examples of transformations are centring with respect to the overall mean, centring with respect to variable (gene) or sample (microarray) means, normalization of variables, scaling by row or column means, and finally the square root and logarithmic transforms. The effect of these transformations radically affects what is plotted and the utility of the resultant plot for a given application.

Many authors standardize or 'norm' the gene expression values for each gene. If this transformation is used for the type of plots described here then the gene points will tend to lie on the circumference of the circle. This is because the variances, which are approximated by the square of the distance of the gene point to the origin, have been set to one, for all genes. Gene points which lie off the circumference have poor fit in lower dimensions.

The standard h-plot centers the gene profile values so their means are zero and scales the singular vectors by the square root of one less than the number of samples. This ensures that the variances and covariances between the genes is reflected in the geometry of the plot. The use of the log transformation is usual with positively skewed microarray gene expression data. If skewed data is used then plots (and analyses) will tend to be dominated by the typical presence of a few very large or very small gene expression values. The log transform has the effect of stretching the lower values close to zero, and sometimes an 'offset' is use to translate the measurements away from zero.

Spectral and singular value decompositions form the basis of many data analysis methods developed for microarray analysis [18-20]. Principal Components analysis (PCA) is well known, but its usefulness for displaying gene expression data depends on using the appropriately scaled and organized gene expression matrix [21]. Other Biplots which use SVD in their algorithms include correspondence analysis (CA) [22] which was developed for frequency tables and Aitchinson's compositional biplot [13] that is most suited to data where the 'variables' or vectors involve an implicit constraint – such as sum. They differ in the scaling applied to the matrix on which the SVD is performed.

Both plots described in this paper have close connections to other methods based on spectral decompositions and singular value decomposition algorithms when used with suitable transformed matrices. However it is only with the factorizations and transformation described in the Methods section that the interpretations in terms of variances and correlations are possible. To maintain these interpretations it is also important to keep the scales on each axis of the display the same, which is not the default in some biplot software.

In this paper we have concentrated on the use of time series plots, because the h-profile plot and covariance biplot was demonstrated on cell cycle data. However other thumbnails in other settings are possible including for example, residuals from statistical and time series models, fitted values from models, smoothed representations, survival curves etc. Another, very important, use of the methodology presented in this paper is the association of annotation information with similar gene expression profiles. Annotation can be added to the profiles, such as chromosome number and/or location, gene ontology information etc. Alternatively suitable thumbnails summarizing annotation information can be displayed at the coordinates described.

Gene selection is important for producing meaningful plots or analyses. The gene selection method demonstrated in this paper has the advantage of providing an estimate of amplitude and its standard error. In this paper we were more interested in selecting genes whose profiles matched a 2 cycle periodic function of any amplitude and to use the plots to explore the profiles further. In a comparison of computational methods for the identification of cell cycle-regulated genes, de Lichtenberg *et al. *[11] concluded that methods that did not take into account the amplitude of the response did not perform well. The placement of the thumbnails in the h-profile plot also provides a visual estimate of the standard deviation of the genes, which was used by de Lichtenberg *et al. *[11] to define regulation. They argued that heavily regulated genes would have large standard deviations, whereas genes without significant regulation would display little deviation from the mean.

If the fit value is not high then the variances and covariances (correlation) will not be well represented and in practice it is often useful to modify the selection criteria, using the visualisation tools demonstrated in this paper in an iterative process.

We have demonstrated the plots here on data generated in order to study cell cycle regulated genes, but the utility of the plots extends beyond these types of applications. In other microarray studies, correlation between gene expression profiles has often been used as a criteria for selecting co-expressed genes. Pearson's product moment correlation coefficient is arguably the most popular measure of similarity between genes used in microarray data analysis. However clustering methods using correlation are often unable to detect subsets of genes which have the same profiles in a subset of the microarrays, but are uncorrelated in other subsets. This is because genes which show highly correlated patterns of expression in one biological state, but not in another (i.e. different subsets of microarrays), may not be highly correlated across the entire data set. The GE-biplot and the h-profile plots facilitate the finding of such subsets.

We have not addressed the question of simultaneous hypothesis testing. Many of the approaches developed to address this challenge are not suited to highly correlated data, and here we are interested in finding correlated gene expression patterns.

## Conclusion

In this paper we demonstrate the usefulness of h-profile plots to simultaneously visualize many gene profiles. By using the coordinates of the h-plot [2,14] to position genes in k-dimensional space, genes with similar gene expression profiles are placed in the same sector, those with high and low variance profiles being separated according to the distance from the origin. Further, positively correlated profiles are placed on the opposite sides of the plot to negatively correlated profiles. Using thumbnail versions of profile plots, the profiles can be readily identified.

Visualization methods are an important component of data analysis, both supporting and being supported by bioinformatic and statistical analyses. The methods described here are easy to implement and could become part of routine analyses (R source code for the GE-Biplot and Gene-plot is available at ). h-Profile plots can be used for detecting novel or unexpected groups of genes with similar profiles, or for displaying profiles following selection of genes, or following application of a machine learning algorithm or a statistical method. They facilitate the finding of genes which have similar or reversed profiles.

## Methods

### The h-plot and covariance Biplot

Gabriel [3] introduced the biplot as a method for displaying the elements of a matrix as inner products of vectors corresponding to the rows and columns of a matrix. Gower and Hand [23] have extended and generalized the ideas of Gabriel. Two earlier descriptions of the biplot for microarray data are Chapman [19] and Pittelkow and Wilson [14]. There is some confusion about the definition of biplots [23] and a variety of biplots have been proposed, some more suited than others to the analysis of microarray data.

Any matrix, **Z**, of rank *r *and size *N*_*c *_by *N*_*g *_can be factored as

**Z **= **CG**^**T**^

where **C **is a matrix of size *N*_*c *_by *r*, and **G **is *N*_*g *_by *r*. Any element of **Z **can therefore be written as *z*_*ij *_= $c_{i}g_{j}^{T}$, where *c*_*i *_is row of **C **and *g*_*j *_is a row of **G**.

The biplot, as described by Gabriel [3], is the plot of all the *N*_*c *_+ *N*_*g *_vectors, *c*_*i*_, *i *= 1, ..., *N*_*c *_and *g*_*j*_, *j *= 1, ..., *N*_*g*_. Originally, both the rows and the columns were represented on the biplot by vectors (lines from the origin or rays), then it became common practice to use the vector representation only for the columns (variables) but, with microarray data, the vector representation is used for the rows (microarrays).

To be practical as a display, *r *would need to be two or three. The matrix of gene expression indices is not, in general, of sufficiently low rank to plot usefully. The usual approach, in this case, is to find a rank *k *approximation to **Z **where *k *is usually two or three. An exact biplot representation of the rank-*k *approximation matrix is known as an 'approximate biplot', but for ease of notation we drop the qualifier 'approximate'.

It is known that one may use the singular value decomposition (SVD) to approximate any rectangular matrix by a matrix of the same size but of lower rank, such that the sums of squares of the differences between the elements of the matrix and its approximation is minimized. Let the SVD of a rank-*r *matrix, **Z**, be **Z **= **U Λ V**^*T *^where **U**, size *N*_*c *_by *N*_*c*_, and **V**^*T*^, size *N*_*g *_by *N*_*g*_, are orthogonal matrices such that **U**^*T*^**U **= **I **and **V**^*T*^**V **= **I**. The notation **I **is used to denote a conformable identity matrix.

The matrix **Λ **of size *N*_*c *_by *N*_*g *_has elements, *λ*_*ij *_= 0, if *i *≠ *j*, and *λ*_*ij *_= *λ*_*i*_, if *i *= *j*. The scalars *λ*_*i *_are ordered such that *λ*_1 _≥ *λ*_2_, ≥ ... ≥ *λ*_*r *_> 0 and are the singular values of **Z **and the positive square roots of the nonzero eigenvalues of **Z**^*T*^**Z **and **ZZ**^*T*^. Since **Z**^*T*^**Z **= **V Λ**^2 ^**V**^*T*^, the columns of **V**, the right singular vectors of **Z**, are also the eigenvectors of **Z**^*T*^**Z**. Similarly it can be shown that the columns of **U**, the left singular vectors of **Z**, are the eigenvectors of **ZZ**^*T*^. A rank *k*, (*k *≤ *r*) approximation to **Z **is given by

$${\hat{Z}}_{k}=U_{k}\Lambda_{k}V_{k}^{T},$$

where **U**_*k *_and **V**_*k *_are matrices comprising the first *k *columns of **U **and **V **respectively and **Λ**_*k *_is a sub matrix of **Λ **formed from the first *k *columns and rows of **Λ**.

Eq. (7), rewritten as

$${\hat{Z}}_{k}=(U_{k}\Lambda_{k}^{\alpha })(\Lambda_{k}^{1-\alpha }V_{k}^{T}),$$

where 0 ≤ *α *≤ 1, can be used to factorize ${\hat{Z}}_{k}$. If **C**_*k *_and **G**_*k *_are defined as **C**_*k *_= **U**_*k *_$\Lambda_{k}^{\alpha }$, and **G**_*k *_= **V**_*k *_$\Lambda_{k}^{1-\alpha }$, then ${\hat{Z}}_{k}=C_{k}G_{k}^{T}$.

The factorization of ${\hat{Z}}_{k}$ into $C_{k}G_{k}^{T}$ is not unique since for any (conformable) invertible matrix **A**, ${\hat{Z}}_{k}$ = (**C**_*k*_**A**) (**G**_*k*_(**A**^*T*^)^-1^)^*T *^= $C_{k}G_{k}^{T}$. The transformations, **A **and (**A**^*T*^)^-1 ^differ only by a scaling. To see this, let the singular value decomposition of the matrix, **A **be as follows **A **= **U**^† ^**Λ**^† ^$V^{{†}^{T}}$, where **U**^† ^and **V**^† ^are k × k orthogonal matrices and Λ^† ^is *diag *($\lambda_{1}^{†},...,\lambda_{k}^{†}$) with $\lambda_{i}^{†}$, the singular values of the decomposition. Then (**A**^*T*^)^-1 ^= **U**^† ^(Λ^†^)^-1 ^**V**^†*T*^. Thus, if **W **is a diagonal matrix of elements *w*_*i*_, *i *= 1, ..., *k *this indeterminacy can be made clear by writing

$$C_{k}=U_{k}\Lambda_{k}^{\alpha }W_{k}$$

and

$$G_{k}=V_{k}\Lambda_{k}^{1-\alpha }W_{k}^{-1}.$$

To ease notation in the following, assume that *k *is given, say 2, and write $\hat{Z}={\hat{Z}}_{k},\hat{C}=C_{k}$ and $\hat{G}$ = **G**_*k*_.

Different scalings affect the position of the points in the plane. If the matrix **Z **is gene centered, i.e. if for each gene, the average gene expression measurement is subtracted from its individual measurements, and in the biplot factorization with *α *= 0 and **W **= $\sqrt{N_{c}}$**I **or **W **= $\sqrt{N_{c}-1}$**I**, the configuration of the gene points is determined from the variance-covariance matrix of the genes. (Or, more correctly, an approximation to the variance-covariance matrix of the genes.)

Given **W **= $\sqrt{N_{c}}$**I **(or **W **= $\sqrt{N_{c}-1}$**I**), $\hat{C}=\sqrt{N_{c}-1}U_{k},{\hat{G}}^{T}=\frac{1}{\sqrt{N_{c}-1}}\Lambda_{k}V_{k}^{T}$, and $\hat{Z}=\hat{C}{\hat{G}}^{T}$, $\hat{G}{\hat{G}}^{T}≃\frac{1}{N_{c}}Z^{T}Z$.

The covariance Biplot uses the coordinates, $\hat{C}$ (microarray points) and $\hat{G}$ (gene points). The h-plot uses the coordinates $\hat{G}$.

Using the geometry of inner products, the juxtaposition of microarrays and genes provides information about the size of the mean corrected gene expression. Any element of $\hat{Z}$ can be calculated as the inner product between the two 2-dimensional row vectors, ${\hat{c}}_{i}$ and ${\hat{g}}_{j}$, as follows;

$$z_{ij}={\hat{c}}_{i}{\hat{g}}_{j}=||{\hat{c}}_{i}||||{\hat{g}}_{j}||\cos({\hat{c}}_{i},{\hat{g}}_{j})$$

where $\cos({\hat{c}}_{i},{\hat{g}}_{j})$ is the cosine of the angle subtended at the origin between the vectors, ${\hat{c}}_{i}$ and ${\hat{g}}_{j}$ and ||·|| denotes length. Thus if the point represented by ${\hat{c}}_{i}$ is close to the point ${\hat{g}}_{j}$ then it can be deduced that the gene represented by the point ${\hat{g}}_{j}$ is relatively up regulated in the microarray represented by the point ${\hat{c}}_{i}$. If ${\hat{g}}_{j}$ is on the opposite side of the plot to ${\hat{c}}_{i}$ then the gene is relatively down regulated on the microarray.

How accurate these reductions are depends on how good the approximation is in the lower ranked space. Two measures of goodness-of-fit for a *k*-dimensional display, which we call *I*_1 _and *I*_2_, are available. They range from zero to one, with a high value assuring close approximation. $I_{1}=\sum_{i=1}^{k}\lambda_{i}^{2}/\sum_{i=1}^{r}\lambda_{i}^{2}$ is an absolute goodness of fit statistic, and $I_{2}=1-{\left\|Z^{\hat{T}}Z-{\hat{G}}^{T}\hat{G}\right\|}^{2}/\left\|Z^{\hat{T}}Z\right\|=\sum_{i=1}^{k}\lambda_{i}^{4}/\sum_{i=1}^{r}\lambda_{i}^{4}$ is a goodness of fit statistic for the variances and covariances between the genes.

An alternative geometric interpretation of the inner product is as the product of the signed length of one of the vectors and the length of the projection of the other vector onto it. Since ${\hat{z}}_{ij}-{\hat{z}}_{ij^{\prime }}={\hat{c}}_{i}^{T}({\hat{g}}_{j}-{\hat{g}}_{j^{\prime }})$, row points can be projected onto a line joining two column points to obtain a ranking of the rows in terms of column differences. Further since ${\hat{z}}_{ij}-{\hat{z}}_{i^{\prime }j}=({\hat{c}}_{i}^{T}-{\hat{c}}_{i^{\prime }}^{T}){\hat{g}}_{j}$ one can project column points onto a row difference vector to estimate the difference in elements between two rows. Since ${\hat{z}}_{ij}-{\hat{z}}_{i^{\prime }j}-{\hat{z}}_{ij^{\prime }}+{\hat{z}}_{i^{\prime }j^{\prime }}=({\hat{c}}_{i}^{T}-{\hat{c}}_{i^{\prime }}^{T})({\hat{g}}_{j}-{\hat{g}}_{j^{\prime }})$ is the interaction between gene *j *and *j' *on chips *i *and *i'*, row difference vectors which lie at right angles to column difference vectors are indicative of no interaction. Further interpretations can be found in [3,13] and [14].

Since log(*z*_*ij*_/*z*_*i'j*_) ≃ ($c_{i}^{T}-c_{i^{\prime }}^{T}$) *g*_*j*_, gene points can be projected onto a chip difference vector to estimate the log ratio of gene expression between two microarrays. Projection of gene points onto a vector joining two microarrays points provides a visual ranking of the genes in terms of their log ratios on the two microarrays.

Let $\hat{G}$ and $\hat{C}$, denote the gene and microarray coordinates respectively. Then the coordinates can be seen as the solutions to the bilinear model,

$$\hat{Z}=\hat{G}{\hat{C}}^{t}+E.$$

There is an indeterminacy in this model which is approached in different ways. Wentzell *et al *[24], for example, use multiple curve resolution methods from chemistry to resolve the indeterminacy in the solution space. Note that Wentzell *et al*'s profiles are another example of profiles to those described here. In the h-plot and covariance-plot, the indeterminacy is resolved so that the inner-products of the gene vectors ($\hat{G}{\hat{G}}^{T}$) approximates the variance-covariance matrix of the genes.

## Authors' contributions

YP conceived, analyzed the data and drafted the manuscript. SW contributed to the statistical methodology and was involved in critical revision of the manuscript. Both authors read and approved the final manuscript.

## Supplementary Material

Additional file 1 Plots of equation 5. *L *ranges from 0 to 3, steps 0.5, with *C *= 1 and *A *= 1. Vertical axis is *z *and horizontal axis shows phase, as described in the text. Click here for file
