# Supplementary figures and images for: h-Profile plots for the discovery and exploration of patterns in gene expression data with an application to time course data
Source: BMC Bioinformatics. 2007 Dec 20;8:486. doi: 10.1186/1471-2105-8-486 (PMC2257978; doi:10.1186/1471-2105-8-486)

**$L = 0$**

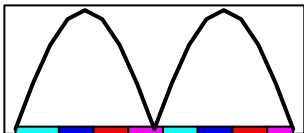

**$L = 0.5$**

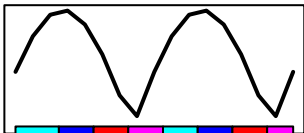

**$L = 1$**

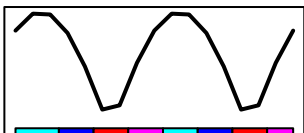

**$L = 1.5$**

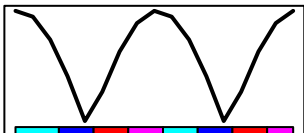

**$L = 2$**

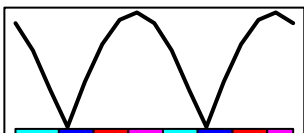

**$L = 2.5$**

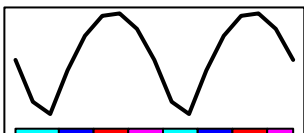

**$L = 3$**

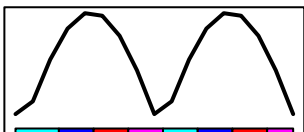

Supplement: Additional file 1 — Plots of equation 5. L ranges from 0 to 3, steps 0.5, with C = 1 and A = 1. Vertical axis is z and horizontal axis shows phase, as described in the text. [file 1471-2105-8-486-S1.pdf]
